# Cognitive behavioural therapy for chronic fatigue and chronic fatigue syndrome: outcomes from a specialist clinic in the UK

**DOI:** 10.1177/0141076820951545

**Published:** 2020-09-15

**Authors:** James Adamson, Sheila Ali, Alastair Santhouse, Simon Wessely, Trudie Chalder

**Affiliations:** 1 Department of Psychological Medicine, Institute of Psychiatry, Psychology and Neuroscience, Kings College London, London SE5 8AF, UK; 2 Persistent Physical symptoms Research and Treatment Unit, South London and Maudsley NHS Foundation Trust, London, SE5 8AZ, UK

**Keywords:** Evidence-based practice, psychotherapy, somatoform disorders, mood disorders (including depression), other psychiatry

## Abstract

**Objectives:**

Cognitive behavioural therapy is commonly used to treat chronic fatigue syndrome and has been shown to be effective for reducing fatigue and improving physical functioning. Most of the evidence on the effectiveness of cognitive behavioural therapy for chronic fatigue syndrome is from randomised control trials, but there are only a few studies in naturalistic treatment settings. Our aim was to examine the effectiveness of cognitive behavioural therapy for chronic fatigue syndrome in a naturalistic setting and examine what factors, if any, predicted outcome.

**Design:**

Using linear mixed effects analysis, we analysed patients' self-reported symptomology over the course of treatment and at three-month follow-up. Furthermore, we explored what baseline factors were associated with improvement at follow-up.

**Setting:**

Data were available for 995 patients receiving cognitive behavioural therapy for chronic fatigue syndrome at an outpatient clinic in the UK.

**Participants:**

Participants were referred consecutively to a specialist unit for chronic fatigue or chronic fatigue syndrome.

**Main outcome measures:**

Patients were assessed throughout their treatment using self-report measures including the Chalder Fatigue Scale, 36-item Short Form Health Survey, Hospital Anxiety and Depression Scale and Global Improvement and Satisfaction.

**Results:**

Patients’ fatigue, physical functioning and social adjustment scores significantly improved over the duration of treatment with medium to large effect sizes (|d| = 0.45–0.91). Furthermore, 85% of patients self-reported that they felt an improvement in their fatigue at follow-up and 90% were satisfied with their treatment. None of the regression models convincingly predicted improvement in outcomes with the best model being (R^2^ = 0.137).

**Conclusions:**

Patients’ fatigue, physical functioning and social adjustment all significantly improved following cognitive behavioural therapy for chronic fatigue syndrome in a naturalistic outpatient setting. These findings support the growing evidence from previous randomised control trials and suggest that cognitive behavioural therapy could be an effective treatment in routine treatment settings.

## Introduction

Fatigue is a ubiquitous symptom that is normally distributed in the population.^1^ For some people, fatigue becomes chronic and starts to affect quality of life. At the more severe end of the spectrum, chronic fatigue syndrome, also known as myalgic encephalomyelitis, is characterised by long-term fatigue, post-exertional malaise and other persistent symptoms such as sleep disturbance, cognitive problems and muscle pain.^2,3^ By definition, chronic fatigue syndrome is associated with marked disability and is associated with reduced participation in social activities, sickness absence and unemployment.^4,5^

There is no consensus on the most accepted diagnostic criteria. The most widely applied case definition is the Centre for Disease Control criteria.^6^ According to Fukuda et al., to meet criteria for a diagnosis of chronic fatigue syndrome, an individual must have self-reported persistent or relapsing fatigue for at least six months, of new or definite onset, that is severe enough to impair occupational, educational, social or personal activities. They must also report four or more of the following symptoms: impaired memory or concentration, sore throat, tender cervical or axillary lymph nodes, muscle pain, multi-joint pain, headaches, unrefreshing sleep and post-exertional malaise lasting for more than one day. These symptoms should last six or more consecutive months and not predate the fatigue.^2^

The most widely used treatments for chronic fatigue syndrome are cognitive behavioural therapy and graded exercise therapy, both of which are recommended for chronic fatigue syndrome by the National Institute for Health and Care Excellence.^7^ There is evidence from systematic reviews and meta-analyses that cognitive behavioural therapy and graded exercise therapy can lead to positive outcomes for patients with chronic fatigue syndrome.^8–10^ A large-scale randomised control trial evaluating cognitive behavioural therapy and graded exercise therapy combined with standard medical care found both of them led to reductions in fatigue and improvements in physical functioning compared to standard medical care alone or a credible therapist matched control, adaptive pacing therapy.^10^

Most of the research has taken place using randomised control trial methodology. However, the complex and rigorous procedures, costs and governance around clinical trials may result in skewed samples, which limit the generalisability of the results. Those who take part in trials may have less co-morbidity and better adherence than those who do not, and staff working on such trials may also differ from those delivering routine clinical treatments.^11^

There is some evidence of positive outcomes from studies that have been conducted outside the confines of a randomised control trial within the context of specialist services for chronic fatigue syndrome. A large cohort study of specialist services in the UK found positive outcomes such as reductions in fatigue, anxiety and depression.^12^ Flo and Chalder conducted a study within routine practice and found that after cognitive behavioural therapy treatment, just under 40% of patients no longer met Oxford or Centers for Disease Control and Prevention criteria for chronic fatigue syndrome, and just under 20% were recovered, similar to rates of recovery reported in the Netherlands.^13^

The positive effects of cognitive behavioural therapy may be maintained long term, regardless of the setting in which the treatment took place (randomised control trial or clinic). Janse et al. conducted a long-term follow-up of four groups of patients with chronic fatigue syndrome who had received cognitive behavioural therapy. Two groups were recruited as part of a randomised control trial and two were clinical cohorts. Fatigue severity and physical functioning improvements were stable up to 18 months following end of treatment. At 18 months to 5 years, one-third of participants were not severely fatigued and almost three-quarters had good levels of physical functioning.^14^

Research suggests that older age has been consistently shown to be a predictor of poor outcomes.^11,12,15^ Furthermore, worse social adjustment, catastrophising and depression all predicted poor outcomes in patients with chronic fatigue syndrome in a specialist clinic.^15^ Baseline physical functioning (36-item Short Form Health Survey) and increased levels of pain predicted poor outcomes in a large study across six specialist units.^12^ However, duration of illness, counter-intuitively, was not a predictor of outcome regardless of setting.^12,16–18^

Most research investigating the effectiveness of treatment for chronic fatigue syndrome has been conducted in the context of a randomised control trial. The aim of this study was to describe the outcomes of people with chronic fatigue syndrome treated at a specialist service with cognitive behavioural therapy. We assessed change over time and also investigated predictors of outcome at the end of treatment. We retrospectively analysed data collected during the previous 14 years of the clinic. We hypothesised that:

1. Fatigue would be reduced, and physical and social functioning improved, after treatment.
2. Older age, higher baseline fatigue, work and social adjustment, anxiety and depression scores and lower physical functioning will be correlated with worse outcome at follow-up.

## Methods

### Participants

Participants were referred to a specialist unit for chronic fatigue syndrome by their general practitioner or by a hospital consultant. This naturalistic study used data retrospectively from patients who were seen in the unit between August 2002 and August 2016. Data were collected from August 2002 to February 2018 inclusive, to include follow-up appointments. All participants were assessed by a specialist during their first appointment with the service. Patients were excluded from the analysis if they had received home-based treatment, if they were still in active treatment at the time of analysis, if they had received a treatment other than cognitive behavioural therapy or if they had not completed a pre-treatment questionnaire measure.

### Ethics

Audit approval was provided by the Psychological Medicine Clinical Academic Group (ID number PPF191115) at the South London and Maudsley Hospital. All patients provided informed written consent.

### Treatment

All patients received individual cognitive behavioural therapy by therapists experienced in treating chronic fatigue syndrome. Therapists received individual supervision on a regular basis with training completed in-house, ensuring adherence to the protocol. Patients are usually offered up to 20 sessions, including follow-up appointments, and receive therapy on a fortnightly basis for 8 to 10 months, depending on interruptions, before transitioning to follow-up appointments.

Treatment was based on the protocol outlined in previous randomised control trials^19^ and has remained fairly consistent across the years. Cognitive behavioural therapy treatment is based on a model which assumes that certain triggers such as a virus and/or stress trigger symptoms of fatigue. Subsequently, symptoms are perpetuated inadvertently by unhelpful cognitive and behavioural responses. After a detailed assessment was carried out, a formulation of the individual's problems was shared with them. Collaboratively patients were then supported with implementing strategies such as monitoring sleep and activity, setting goals, establishing routines, sleep hygiene, avoiding boom and bust cycles of behaviour, reducing excessive avoidance behaviour, tackling stress and addressing unhelpful beliefs which may be interfering with helpful changes. When relevant and appropriate, early childhood trauma will often be included in the formulation of patients' difficulties and may be referred to when patients are tackling unhelpful beliefs or schemas. Difficulties in emotion regulation linked to holding beliefs that expressing negative emotions to oneself or to others is unacceptable are also addressed if such issues become apparent during therapy.

### Measures

Participants were asked to complete questionnaire measures at the start of treatment, at sessions 4 and 7, at discharge and at three-month follow-up. The questionnaires consisted of the following:

#### Demographics

Patients were asked their age, gender, ethnicity, duration of illness as well as duration, intensity and type of fatigue, in line with Oxford and Centre for Disease Control criteria for chronic fatigue syndrome.^2,3^

Fatigue was measured using the Chalder fatigue scale. This has been shown to be reliable and valid.^20,21^ It consists of 11 items, each of which has four possible response options, ranging from ‘less than usual’ to ‘much more than usual’. Either a continuous scoring system (0, 1, 2, 3) or a bimodal scoring (0, 0, 1, 1) method can be used for each item which is summed to obtain a total score. A higher score is associated with greater fatigue severity. Using the bi-modal scoring system a cut-off score of 4 or more can be used as an indicator of fatigue caseness.^20^

Social adjustment was measured using the Work and Social Adjustment Scale, which has been validated in patients with chronic fatigue syndrome.^22^ This is a five-item scale where each item receives a score between 0 and 8 and the items are summed to obtain a score out of 40. A higher score indicates greater impairment, i.e. worse social adjustment.

Physical functioning was measured using the 36-item Short Form Health Survey, physical functioning subscale. This consists of 10 items which are summed to give a total out of 100. A higher score indicates better physical functioning. This scale has been shown to be valid and reliable.^23^

Anxiety and Depression was measured using the Hospital Anxiety and Depression Scale.^24^ The Hospital Anxiety and Depression Scale is a 14-item self-report questionnaire with seven questions assessing severity of each disorder-related symptom over the past week. Higher scores indicate more severe symptomology with a maximum possible score of 21 and a cut-off score of 8.^25^ The Hospital Anxiety and Depression Scale is assessed only at the start and end of treatment.

Global improvement was assessed on a six-point scale, ranging from 1 (very much better) to 6 (very much worse). In line with previous randomised control trials, the responses were also coded into a dichotomous variable: 1 = improved (very much better and much better); and 0 = not improved (little better to very much worse).^10^

Satisfaction with treatment was rated on a seven-point scale ranging from 1 (very satisfied) to 7 (very dissatisfied).

#### Missing data

If a patient was missing 25% or less of the data from any questionnaire, a pro-rated score was calculated (this used the mean of the remaining items from the same individual to calculate a prorated score).

### Analysis

Data management, descriptive statistics and analysis was conducted in SPSS version 24.^26^ Alpha was set at *p* < 0.05. In addition, effect size of change in outcomes from start to end of treatment was estimated using Cohen's d (|d|).^27^ The effect size estimates were interpreted as small (|d| ≥ 0.2), medium (|d| ≥ 0.5) and large (|d| ≥ 0.8). Demographic characteristics of the sample were described using measures of central tendency.

#### Drop-out

Patients who did not complete discharge or follow-up measures were considered to have dropped out from treatment. We compared those who dropped out to those who completed treatment using independent samples t-tests to compare demographic and baseline Chalder Fatigue Questionnaire, 36-item Short Form Health Survey, and Work and Social Adjustment Scale.

#### Change in main outcomes over time

We measured the effect of time on Chalder Fatigue Questionnaire and Work and Social Adjustment Scale scores over all the observed time points using linear mixed models (compound symmetry model) with time as the main predictor. For the 36-item Short Form Health Survey, due to low numbers at sessions 4 and 7, we analysed the effect of time over start, discharge and three-month follow-up. Post-hoc paired samples t-tests (Bonferroni corrected for multiple comparisons)^28^ were used to assess changes between each time point for each outcome. Since anxiety and depression were not direct targets for the cognitive behavioural therapy intervention, we were only interested if there was a clinically significant change in patients' self-reported symptomology, i.e. patients moved from case to non-case, according to the Hospital Anxiety and Depression Scale. We assessed how many people had shown global improvement and rates of satisfaction using descriptive statistics. Deterioration was assessed by number deteriorated, i.e. how many became a fatigue case (score > 4) that were not a case at the start of treatment and how many people showed a worsening of fatigue (two-point increase out of 33).

#### Predictors of outcome

Predictors of outcome were assessed using the following variables: age, ethnicity (dichotomous), illness duration, and baseline fatigue, Work and Social Adjustment Scale, 36-item Short Form Health Survey, and Hospital Anxiety and Depression Scale. Multiple linear regression was used to analyse predictors of improvement with Chalder Fatigue Questionnaire, 36-item Short Form Health Survey and Work and Social Adjustment Scale improvement scores (difference in scores over time) used as dependent variables. For those patients without end of treatment scores, the nearest follow-up score was used to compute an endpoint and was defined as a computed follow-up score.

## Results

### Participants

Nine hundred and ninety-five participants were included in the analysis. All participants were treated for chronic fatigue syndrome at the National Persistent Physical Symptoms Research and Treatment Unit. All met National Institute for Health and Care Excellence criteria for chronic fatigue syndrome.^7^ According to self-reported accounts of their symptoms, 754 (76%) participants met Oxford criteria for chronic fatigue syndrome and 518 (52%) met Centre for Disease Control criteria for chronic fatigue syndrome. Nine hundred and fifteen (92%) patients reported both physical and mental fatigue with 67 (7%) reporting either physical or mental fatigue.

Two hundred and sixty (26%) participants were male and 729 (73%) were female. Six (1%) did not state their gender. Participants were aged between 18 and 74 years (mean age 39.45 years, standard deviation 11.5); 784 (78.7%) participants were white. Participants had been ill for a mean duration of 6.65 years (standard deviation 6.48); 437 (44%) were married or living together and 423 (43%) were single. Moreover, 228 (23%) were educated to school level (GCSE/O Level) with 683 (69%) educated to university level (undergraduate).

### Number of sessions

All participants received cognitive behavioural therapy with a mean duration of 12 sessions (standard deviation 4.9; range 1–30). The majority had 9–16 sessions (67.1%), 8.2% had 4 or less and 11.7% had 16 or more sessions.

### Drop-out

Drop-out was defined as those patients who did not complete any questionnaires at the end of treatment or at any follow-up, and was 31% in this naturalistic setting. Reason for drop-out was only recorded since 2007, and therefore, we have no data from 2002 to 2007. Data are presented for those with drop-out information (n = 140) with reasons for frequencies greater than 5%. Frequencies less than 5% are combined in ‘other’ and include: only funded for small amount of sessions; patient did not engage with treatment; and referred to another service (see Table 1).

**Table 1.** Reason for drop-out.

Reason	*n (%)*
Dropped out with no contact	29 (21)
Discontinued treatment (did not want treatment)	21 (15)
Cancelled numerous appointments and was discharged	14 (10)
Moved away from area	14 (10)
Felt better and discontinued treatment	7 (5)
Treatment stopped due to other co-morbid problem	7 (5)
Had to stop treatment due to personal circumstances (e.g. life events)	7 (5)
Other	41 (29)
Total	140

### Drop-out vs. stay in

There were no statistically significant differences in demographic characteristics between those who dropped out from treatment and those who completed. We did find a statistically significant difference in self-reported physical functioning at baseline between the groups (t(763) = −3.74, *p* < 0.001) with those that dropped out reporting lower physical functioning scores with a mean difference of −7.38. Furthermore, those who dropped out had more severe Work and Social Adjustment Scale scores at baseline (t(987) = 2.48, *p* < 0.05) with a mean difference of 1.55. However, no difference in level of fatigue was observed (t(975) = 1.61, *p*>0.05). Those who dropped out also had higher scores on self-reported depression symptoms at baseline (t(981) = 2.35, *p* < 0.05) but no difference in anxiety scores (t(978) = 1.4, *p*>0.05).

### Change in main outcomes over time

Estimated marginal means and standard errors for Chalder Fatigue Questionnaire, 36-item Short Form Health Survey, and Work and Social Adjustment Scale are shown in Table 2.

**Table 2.** Estimated marginal means and standard errors for main outcomes over time.

Measure	Assessment	*n*	Mean	*SE*	95% Confidence interval	
					Lower bound	Upper bound
CFQ	Start	977	24.19	0.25	23.69	24.68
	Session 4	392	19.45	0.36	18.75	20.15
	Session 7	380	18.62	0.36	17.91	19.32
	Discharge	581	17.67	0.31	17.07	18.26
	Follow-up	503	18.60	0.32	17.96	19.23
SF-36	Start	768	47.60	0.95	45.73	49.46
	Discharge	441	57.50	1.07	55.40	59.61
	Follow-up	404	58.51	1.10	56.36	60.67
WSAS	Start	989	25.04	0.31	24.43	25.65
	Session 4	395	22.91	0.39	22.15	23.67
	Session 7	382	21.41	0.39	20.65	22.18
	Discharge	582	19.49	0.35	18.80	20.17
	Follow-up	507	19.12	0.36	18.41	19.83

WSAS: Work and Social Adjustment Scale; SF-36: Short Form Health Survey, Physical Functioning Subscale; CFQ: Chalder Fatigue Questionnaire.

**Figure 1.**
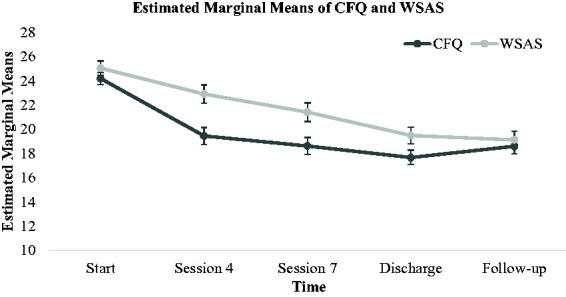
Estimated marginal means for both Chalder Fatigue Questionnaire (CFQ) and Work and Social Adjustment Scale (WSAS) scores across all observed time points.

Using a linear mixed effects model, we can note that there was a significant main effect of time on patients Chalder Fatigue Questionnaire scores, F(4,2069) = 150.88, *p* < 0.001 with Chalder Fatigue Questionnaire significantly improving over time: start of treatment M = 24.20 (standard deviation = 6.95); end of treatment M = 17.42 (standard deviation = 8.83). The effect size was large (|d| = 0.91) with a mean difference of 6.52 [95% confidence interval (5.65–7.39)]. The largest change appears to happen between the start of treatment and session 4 [mean difference = 4.74, 95% confidence interval (3.73–5.75)] with subsequent time point differences not meeting significance, despite main effect being significant. Scores across all time points are displayed in Figure 1.

Due to low numbers of patients completing 36-item Short Form Health Survey at the fourth and seventh sessions, we analysed the main effect of time using three time points (start, discharge and three-month follow-up). The main effect of time was significant (F(2,873) = 111.16, *p* < 0.001) with the 36-item Short Form Health Survey significantly improving over treatment: start of treatment M = 47.81 (standard deviation = 25.74); end of treatment M = 59.56 (standard deviation = 27.3). The effect size was medium (|d| = 0.45) with a mean difference of 9.91 [95% confidence interval (7.94–11.88)]. Treatment improvements appear to be sustained at follow-up, with there being no significant difference in scores between end of treatment and three-month follow-up (mean difference = 1, *p* = 0.82).

There was a significant main effect of time on patients’ Work and Social Adjustment Scale scores, F(4,1961) = 155.75, *p* < 0.001 with Work and Social Adjustment Scale significantly improving over time: start of treatment M = 25.02 (standard deviation = 8.99); end of treatment M = 19.14 (standard deviation = 10.71). The effect size was medium (|d| = 0.61) with a mean difference of 5.55 [95% confidence interval (4.79–6.32)]. Improvement in patients' scores was statistically significant across each time point from start to end of treatment (*p* < 0.001) and then treatment improvements were sustained at follow-up with the difference in scores from discharge to follow-up being non-significant (mean difference = 0.37, *p* = 1). Means with 95% confidence interval are displayed in Figure 1.

### The presence of anxiety and depression

Table 3 shows Hospital Anxiety and Depression Scale caseness for participants with complete data (using cut-off score of 8).

**Table 3.** HADS caseness for participants with complete data (using cut-off score of 8).

Measure	Caseness	Start	Discharge	Follow-up
		*n* (%)	*n* (%)	*n* (%)
HADS-A	Case	257 (65)	204 (52)	204 (52%)
	Non-case	136 (35)	189 (48)	189 (48%)
	Total	393		
HADS-D	Case	215 (55)	140 (36)	161 (41%)
	Non-case	178 (45)	253 (64)	232 (59%)

HADS-A: Hospital Anxiety and Depression Scale, Anxiety Subscale; HADS-D: Hospital Anxiety and Depression Scale, Depression Subscale.

### Global improvement

Patients largely self-reported that they saw an improvement in their fatigue at discharge, with 87% reporting that they felt at least a little better; only 2.5% felt like they were worse off. At three-month follow-up, 84% reported at least some improvement and only 5% reporting feeling worse. A full report of patient responses at both time points is displayed in Table 4. Using previous randomised control trials’ methodology of dichotomosing global improvement scores, we see a 53% global improvement at discharge and 56% at three-month follow-up.

**Table 4.** Self-reported global improvement at discharge and follow-up, for participants with complete data.

Outcome	Discharge	Follow-up
	*n* (%)	*n* (%)
Very much better	67 (18)	74 (20)
Much better	129 (35)	133 (36)
A little better	124 (34)	102 (28)
About the same	35 (10)	38 (10)
A little worse	8 (2)	13 (4)
Very much worse	2 (0.5)	5 (1)
Total	365	365

### Deterioration in fatigue

According to patients' self-reported Chalder Fatigue Questionnaire scores, a large majority (72%) of patients reported a significant improvement in fatigue, with at least a two-point decrease on the measure. Some patients reported at least a two-point increase in fatigue (16%) indicating a deterioration, according to the measure. The final 12% reported little or no change in fatigue.

### Satisfaction with treatment

Patients were largely very satisfied with their cognitive behavioural therapy treatment with over 90% of patients rating their satisfaction as at least slightly satisfied and 45% saying they were very satisfied. Less than 10% of patients rated their satisfaction with treatment as dissatisfied.

### Predicting outcome

A multiple linear regression was used to predict improvement in Chalder Fatigue Questionnaire, Work and Social Adjustment Scale, and 36-item Short Form Health Survey scores, with improvement defined as the difference between patients’ computed follow-up scores and their start of treatment scores. All participants, except three, had either a discharge or a three-month follow-up score and this represents the computed follow-up score. For the three that had neither, we took the scores from the closest session to discharge we had. A significant regression equation was found for Chalder Fatigue Questionnaire improvement scores (F(8,436) = 8.65, *p* < 0.001), with an R^2^ of 0.137. However, only baseline Chalder Fatigue Questionnaire was positively correlated more than r = 0.3 with Chalder Fatigue Questionnaire improvement and accounts for the majority of the model. We also found a significant regression equation for 36-item Short Form Health Survey improvement scores (F(8,430) = 6.18, *p* < 0.001), with an R^2^ of 0.103. Only baseline 36-item Short Form Health Survey had a notable positive correlation of r = 0.2. Finally, we found a significant regression equation for Work and Social Adjustment Scale improvement scores (F(8,438) = 7.63, *p* < 0.001), with an R^2^ of 0.122. As above, the highest correlated variable with Work and Social Adjustment Scale improvement scores was baseline Work and Social Adjustment Scale scores being positively correlated, r = 0.18.

## Discussion

This naturalistic outcome study investigated the impact of individual cognitive behavioural therapy on patients' self-reported fatigue and physical functioning, after outpatient treatment for chronic fatigue syndrome. The cognitive behavioural therapy intervention led to significant improvements in patients’ self-reported fatigue, physical functioning and social adjustment. Medium to large effect size improvements were observed across all measures between the start and end of treatment (0.45 to 0.91). Interestingly, initial gain in fatigue between the start of treatment and session 4 was where we saw the largest improvement in fatigue and Work and Social Adjustment Scale scores. Furthermore, 72% of participants improved at least two points on the Chalder fatigue scale with 29% of participants scoring below cut-off and therefore becoming a non-case. This is in line with previous findings from Stahl et al., who reported significant improvements in both Chalder Fatigue Questionnaire and Work and Social Adjustment Scale after cognitive behavioural therapy treatment.^29^ However, the present findings differ in that the previous study included Chalder Fatigue Questionnaire measures at only two time points whereas the current study used multiple time points throughout treatment, giving an indication as to when change happened. In this large cohort, changes occurred within the first four sessions. In both studies, improvements were maintained after discharge and into follow-up on all three measures, indicating that the treatment effects were maintained.

Similarly to previous large randomised control trials, these findings suggest cognitive behavioural therapy may be an effective intervention to target fatigue, physical functioning and social adjustment in patients with chronic fatigue syndrome.^10^ By dichotomising self-reported global improvement, 50% of patients in the present study reported feeling much better or very much better. This is in line with previous results from Quarmby et al., in which they found a global improvement score of 57% in routine care,^11^ and Flo and Chalder,^15^ who reported a 60.8% global improvement, using the same methodology.

Reassuringly, we did not find any age or ethnic differences in treatment improvement outcomes across Chalder Fatigue Questionnaire, 36-item Short Form Health Survey, and Work and Social Adjustment Scale. Neither did we find any differences in age, ethnicity, marital status or illness duration for those who dropped out compared to those who completed treatment. We were unable to find any predictors of outcome so cannot say with any certainty who will do well in treatment.

## Limitations

This naturalistic study had high ecological validity. However, the lack of a control condition limits us from drawing any causal inferences, as we cannot be certain that the improvements seen are due to cognitive behavioural therapy alone and not any other extraneous variables. Furthermore, therapist effects were not considered, due to lack of power and results from our previous study in the same setting which suggested therapist effects were minimal.^30^ Future studies should include a waiting list control sample within naturalistic settings to address these issues.

The drop-out rate was 31%, although large meta-analysis reviews found drop-out rates for cognitive behavioural therapy studies range from 0% to 42% depending on study design and definition of drop-out,^9^ suggesting that our drop-out rate was not unusually high. Those who dropped out were more likely to have lower physical functioning, higher Work and Social Adjustment Scale scores and higher depression scores. This suggests that there may have been some bias in the data, in that those who completed treatment may not represent all patients who access cognitive behavioural therapy treatment for chronic fatigue syndrome.

In conclusion, these results demonstrate that cognitive behavioural therapy delivered in a naturalistic setting could lead to positive changes in fatigue, physical functioning and social adjustment. Furthermore, many patients no longer meet diagnostic criteria. Clinics should assess outcomes routinely and report on change in naturalistic settings. Future studies should consider a wait list control and explore other variables which may moderate the treatment effect.

## Declarations

### Guarantor

TC.
